# Novel Riboswitch Ligand Analogs as Selective Inhibitors of Guanine-Related Metabolic Pathways

**DOI:** 10.1371/journal.ppat.1000865

**Published:** 2010-04-22

**Authors:** Jérôme Mulhbacher, Eric Brouillette, Marianne Allard, Louis-Charles Fortier, François Malouin, Daniel A. Lafontaine

**Affiliations:** 1 Département de biologie, Faculté des sciences, Université de Sherbrooke, Sherbrooke, Québec, Canada; 2 Département de microbiologie et d'infectiologie, Faculté de médecine et sciences de la santé, Université de Sherbrooke, Sherbrooke, Québec, Canada; Children's Hospital Boston, United States of America

## Abstract

Riboswitches are regulatory elements modulating gene expression in response to specific metabolite binding. It has been recently reported that riboswitch agonists may exhibit antimicrobial properties by binding to the riboswitch domain. Guanine riboswitches are involved in the regulation of transport and biosynthesis of purine metabolites, which are critical for the nucleotides cellular pool. Upon guanine binding, these riboswitches stabilize a 5′-untranslated mRNA structure that causes transcription attenuation of the downstream open reading frame. In principle, any agonistic compound targeting a guanine riboswitch could cause gene repression even when the cell is starved for guanine. Antibiotics binding to riboswitches provide novel antimicrobial compounds that can be rationally designed from riboswitch crystal structures. Using this, we have identified a pyrimidine compound (PC1) binding guanine riboswitches that shows bactericidal activity against a subgroup of bacterial species including well-known nosocomial pathogens. This selective bacterial killing is only achieved when *guaA*, a gene coding for a GMP synthetase, is under the control of the riboswitch. Among the bacterial strains tested, several clinical strains exhibiting multiple drug resistance were inhibited suggesting that PC1 targets a different metabolic pathway. As a proof of principle, we have used a mouse model to show a direct correlation between the administration of PC1 and the reduction of *Staphylococcus aureus* infection in mammary glands. This work establishes the possibility of using existing structural knowledge to design novel guanine riboswitch-targeting antibiotics as powerful and selective antimicrobial compounds. Particularly, the finding of this new guanine riboswitch target is crucial as community-acquired bacterial infections have recently started to emerge.

## Author Summary

During the last 30 years, bacterial resistance to antibiotics has become a major problem. This situation is partly because today's antibiotics are mainly based on a limited selection of chemical scaffolds, which makes it easier for bacterial pathogens to quickly develop resistance against new drug derivatives. This recurrent problem of multiple drug resistance implies a constant need to search for novel microbial targets and to modulate their activity using artificial molecules. Riboswitches are newly discovered gene regulatory elements that represent attractive targets for antimicrobial drugs. Riboswitches are RNA structures located in untranslated regions of messenger RNAs that regulate the expression of genes involved in the transport and metabolism of small metabolites. We have identified a new antibiotic specifically targeting riboswitches found in a subgroup of bacteria including *Staphylococcus aureus* and *Clostridium difficile,* which are nosocomial pathogens responsible for a significant mortality rate in hospitals, and increased health care costs. The riboswitch controls the expression of *guaA* that appears essential for virulence in the mammalian host. A murine model was used as a proof of principle to show that such an antibiotic could inhibit the growth of *S. aureus* in a mammal. Our work provides new insights into the discovery and design of novel antimicrobial agents against bacterial pathogens.

## Introduction

Multiple drug resistance (MDR) has been a growing problem during the last decade, partly due to excessive use of antibiotics in human medicine and food animal production. MDR also stems from the fact that drug design has been largely based on limited chemical scaffolds leaving an opportunity for pathogens to circumvent antibiotic action mechanisms [1]. *Staphylococcus aureus* and *Clostridium difficile* are nosocomial pathogens responsible for a significant mortality rate in hospitals and increased health care costs [2]. Recently, community-acquired methicillin-resistant *S. aureus* (MRSA) infections have emerged and are commonly responsible for skin and soft-tissue infections that may rapidly evolve in severe and life-threatening infections [3], [4]. Moreover, some emerging clones were shown to be resistant to vancomycin, which is considered as the last chance antibiotic [5]. The pathogen *C. difficile* has also dramatically increased the hospital-associated deaths in recent years due to the MDR emergence and spreading of the hypervirulent and high toxin-producing strain BI/NAP1/027 [4], [5], [6]. This particular strain is spreading in North America and Europe with currently little therapeutic solutions besides the use of metronidazole and vancomycin, which are increasingly associated with relapses and poor treatment outcome [7].

Previous attempts to discover alternative antibacterial drugs targeting RNA were mainly based on a fortuitous interaction between an exogenous ligand and its RNA target [1], [8], [9], [10]. Metabolite-responsive riboswitches represent a novel solution to MDR since they could be considered as antimicrobial targets when agonistic ligands are employed as demonstrated for lysine, thiamine pyrophosphate (TPP), flavin mononucleotide (FMN) and guanine responsive riboswitches [1], [11], [12], [13], [14], [15]. In the case of lysine and TPP riboswitches, previously described ligand analogs were reported to have a multitude of cellular effects in addition to inhibition of gene expression via riboswitch binding [16], [17], [18], [19]. Pleiotropic effects were also observed for compounds targeting the guanine riboswitch and at least one analog was reported to be possibly incorporated in DNA during replication [15], [20]. Thus, while it is of interest to select antibiotics that are chemically distinct from natural ligands to avoid cellular efflux or chemical modification, it is important to consider that these chemical differences will potentially help avoid patient toxicity due to off-target binding. It is also important that the antibiotic provokes a bacteriostatic or bactericidal effect either by targeting a single gene, or a collection of genes, that is necessary for growth, or essential for bacterial survival or virulence. Thus, because modified pyrimidines can specifically bind the purine riboswitch with affinities in the low nanomolar range [21], they make excellent candidates to target purine riboswitches which are likely potent drug targets given their role in regulating purine metabolic pathways (Figure S1). For instance, the inactivation of the *E. coli* GMP synthetase *guaA* leads to guanine auxotrophy [22] whereas the inactivation of the *B. subtilis* IMP dehydrogenase *guaB* is lethal [23]. Here we show that the guanine riboswitch in *S. aureus* and *C. difficile* controls the expression of *guaA* and that this gene appears essential for virulence in a murine model.

## Results

### Pyrimidine-based antibiotics modulate the guanine riboswitch activity

**Figure 1:**
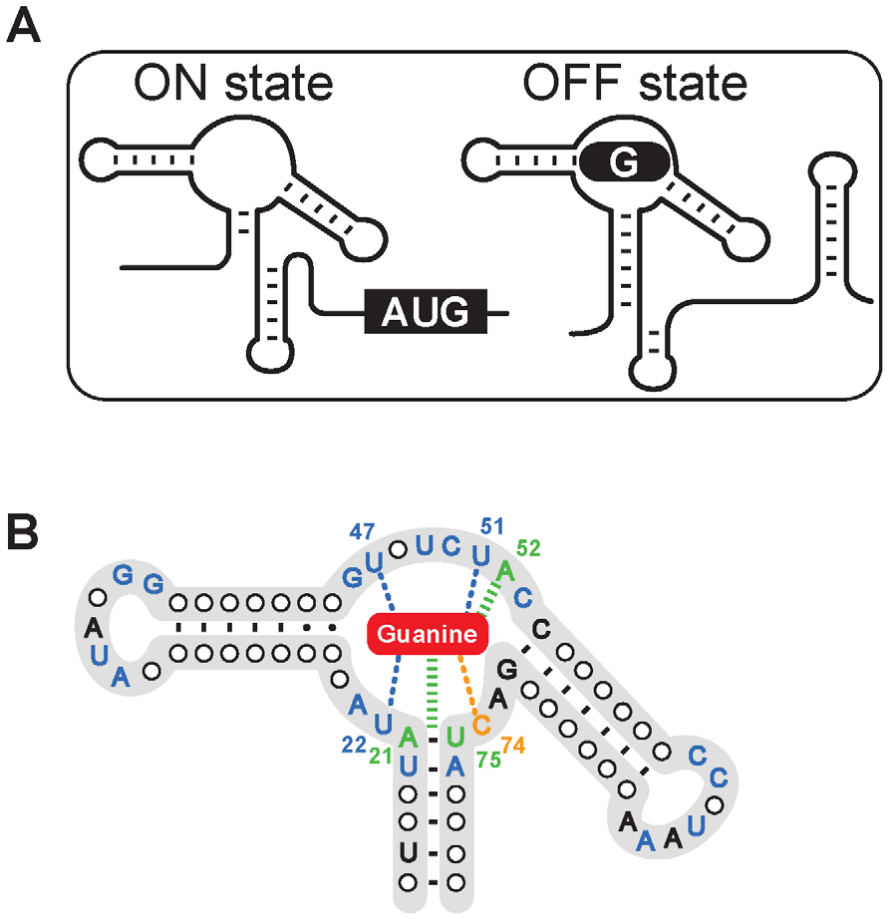
The structure of the guanine riboswitch. (A) Scheme representing the guanine riboswitch secondary structures in absence (ON state) or in presence (OFF state) of guanine. The formation of a guanine-riboswitch complex results in the adoption of an intrinsic terminator element that prematurely stops transcription. (B) Consensus sequence and secondary structure of the guanine riboswitch aptamer. Nucleotides indicated in blue, orange and green represent nucleotides that are conserved >90% and those colored in black are conserved >80% [28]. Nucleotides and lines in blue and in green indicate interactions with the ligand via hydrogen bonding and base stacking, respectively. The cytosine 74 which confers ligand binding specificity is shown in orange and the bound guanine is shown as a red rounded rectangle.

Guanine-sensing riboswitches are members of the purine riboswitch class, which also comprises adenine and 2′-deoxyguanosine [24]. The guanine riboswitch negatively regulates transcription elongation at high guanine concentration in *Bacillus subtilis* [25] (Figure 1A). The guanine aptamer is organized around a three-way junction connecting three helices, in which a critically important nucleotide is involved in a Watson-Crick base pair interaction with the bound ligand [25] (Figure 1B). The ligand binding site contains a cavity in which the metabolite is completely surrounded by RNA contacts suggesting that most atomic positions are important for the formation of the native ligand-RNA complex [26], [27]. By using appropriate aminopyrimidines, it is also possible to recreate the correct network of hydrogen bonds required to ensure proper complex formation as previously shown for the adenine riboswitch [21]. Thus, by taking advantage of the fact that purine riboswitches efficiently bind pyrimidines, it may be possible to design novel antibiotics that bind to guanine riboswitches and therefore inhibit bacterial growth.

**Figure 2:**
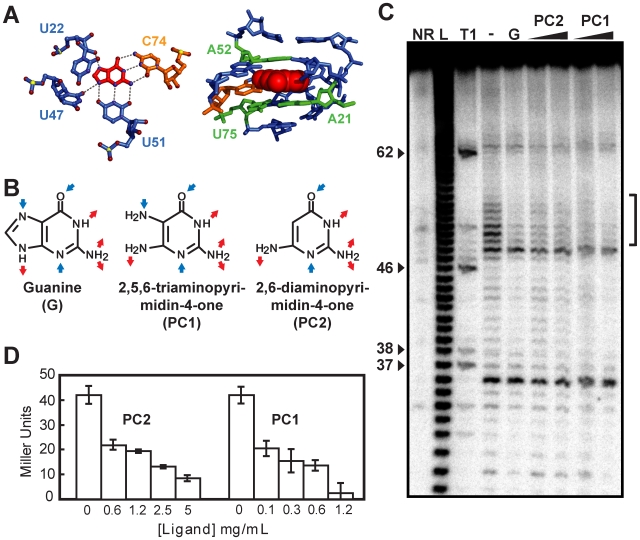
Guanine riboswitch agonists can be used to modulate gene expression. (A) Hydrogen bonds (left panel) and stacking interactions (right panel) formed between the bound guanine and the guanine riboswitch [26], [27]. Oxygen, nitrogen, and phosphorus atoms are in red, blue, and yellow, respectively (left panel). Nucleotides follow the color scheme used in Figure 1B. Figures were prepared using PyMol (DeLano Scientific, San Francisco, CA, USA). (B) Molecular recognition features for guanine (G) and predicted ones for PC1 and PC2. Blue and red arrows represent hydrogen bond acceptors and donors, respectively. (C) In-line probing assays of the *B. subtilis xpt* riboswitch in the absence (−) or in the presence (+) of 1 µM guanine (G), and 1 µM or 10 µM for both PC1 and PC2. Sites of substantial ligand-induced protections (positions 49–54) are assigned on the right by a vertical bracket. Lanes NR, L and T1 correspond to molecules that were not reacted or that were partially digested by alkali or by RNase T1, respectively. Guanines are identified on the left as molecular weight markers. (D) The beta-galactosidase activity of a *xpt-lacZ* transcriptional fusion construct integrated in the genome of *B. subtilis* by recombination was assayed after 4 h of growth at 37°C in minimal medium in presence of the indicated ligand concentrations. Each experiment was performed three times and the average as well as standard deviations are shown.

Pyrimidine-based molecules that could fit into the guanine riboswitch aptamer binding site were selected based on molecular modeling of crystal structures [26], [27] (Figure 2A). Using this approach, we identified two pyrimidine compounds 2,5,6-triaminopyrimidin-4-one (PC1) and 2,6-diaminopyrimidin-4-one (PC2) that satisfied defined criteria such as geometrical constraint, hydrogen bonding pattern and molecule planarity (Figure 2B). As opposed to guanine, PC1 and PC2 lack one aromatic ring, making them electronically distinct from guanine despite their similarity to guanine in terms of H-bond donating and accepting potential. Next, using the established in-line probing assay [25], [28], we monitored PC1/PC2-induced riboswitch conformational changes (Figure 2C). In absence of ligand, several cleavage products that map to previously reported single-stranded regions were observed [25], [28]. However, a cleavage reduction consistent with a reorganization of the structure upon ligand binding was observed in the core domain in presence of guanine (Figure 2C). In-line probing assay with PC1 and PC2 instead of guanine showed an identical cleavage pattern for both pyrimidine compounds and guanine, suggesting that the core is reorganized similarly in presence of these compounds, consistent with the recently reported pyrimidine-bound riboswitch crystal structure [21].

To determine whether PC1 and PC2 repress gene expression, we transformed *B. subtilis* with transcriptional fusions in which a guanine riboswitch was fused to a *lacZ* reporter gene (Figure 2D). When cells were grown in minimal medium with increasing concentrations of PC1 and PC2, beta-galactosidase activity was clearly repressed in a dose-dependent manner suggesting a modulation of the guanine riboswitch gene regulation by both molecules. We also performed growth inhibition experiments using various concentrations of both PC1 and PC2 (Figure S2). While growth inhibition was observed in minimal medium, no such inhibition was observed using a richer medium such as cation-adjusted Muller-Hinton broth (CAMHB). This selective growth inhibition can be explained by PC1/PC2 inhibiting the biosynthesis or transport of essential metabolites, which are present in CAMHB but not in minimal medium. For instance, it was recently shown that guanine-related compounds can only inhibit *B. subtilis* growth in a minimal medium but not in Luria broth; the growth inhibition was partly attributed to the riboswitch-mediated repression of *de novo* purine synthesis [15].

### PC1-dependent bacterial growth inhibition requires *guaA* to be riboswitch-regulated

**Figure 3:**
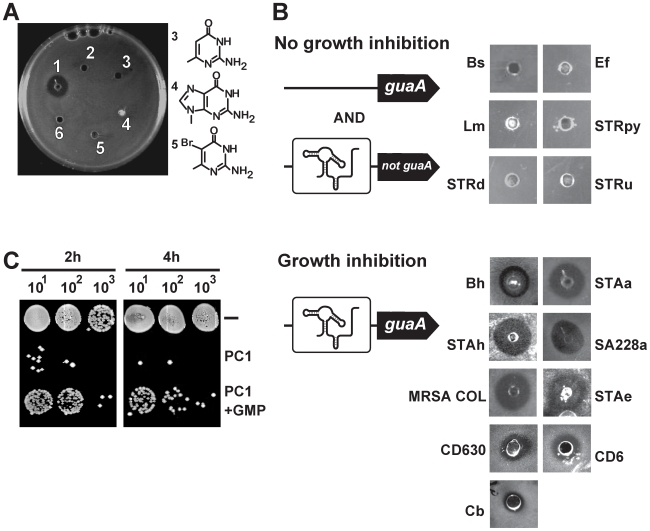
*S. aureus* growth inhibition requires *guaA* to be under a riboswitch control. (A) Antibiograms performed on the *S. aureus* ATCC 29213 strain using 75 µg of PC1 (1), PC2 (2), 2-amino-4-hydroxy-6-methylpyrimidine (3), 9-methyl guanine (4), 5-bromo-6-methyl pyrimidine (5) and PBS as control (6). Chemical structures of PC1 and PC2 are shown in Figure 2B. (B) PC1 antibiograms using bacterial strains with guanine riboswitches. While PC1-insensitive strains do not have *guaA* under a riboswitch control (Bs: *Bacillus subtilis*, Ef: *Enterococcus feacium*, Lm: *Listeria monocytogenes*, STRpy: *Streptococcus pyogenes*, STRd: *Streptococcus dysgalactiae* and STRu: *Streptococcus uberis*), PC1-sensitive strains control the *guaA* gene expression via a riboswitch mechanism (Bh: *Bacillus halodurans*, STAa: *S. aureus* ATCC 29213, STAh: *S. haemolyticus*, SA228a: *S. aureus* resistant to beta-lactam, erythromycin, ciprofloxacin, gentamicin and tetracycline but susceptible to vancomycin, MRSA COL: methicilin resistant *S. aureus* COL, STAe: *S. epidermidis,* Cb: *Clostridium botulinum*, CD630: *C. difficile* strain 630, CD6: *C. difficile* representing the hypervirulent NAP1/027 strain). (C) Influence of GMP on PC1 bacterial growth inhibition. Spots from serial dilutions of *S. aureus* cultures in cation-adjusted Muller-Hinton broth (CAMHB) in absence or presence of 600 µg/mL PC1 or 600 µg/mL PC1 supplemented with 100 µM GMP.

The *S. aureus* ATCC 29213 genome contains a unique guanine riboswitch located immediately upstream of the *xpt* gene (Figure S3). Very interestingly, RT-PCR experiments identified that the riboswitch controls a four-gene operon consisting of *xpt*, *pbuX*, *guaB* and *guaA*, thus placing *guaA* and *guaB* under the control of a riboswitch in *S. aureus* (Figure S3). To determine if PC1 and PC2 have antibiotic activities by targeting the guanine riboswitch in *S. aureus*, we performed antibiograms with PC1 and PC2 as well as with three additional molecules having similar structures (compounds 3, 4 and 5). While compounds 3 and 5 are structurally very close to PC1 and PC2, compound 4 is a guanine analog (Figure 3A). Surprisingly, among the five compounds tested, only PC1 inhibited bacterial growth in Muller-Hinton agar, which is consistent with its ability to modulate riboswitch gene expression in *B. subtilis* (Figure 2D). The absence of PC2 antibiotic activity is consistent with the ∼5-fold lower PC2-mediated gene expression modulation in *B. subtilis*, which may result from the lower number of riboswitch-ligand interactions (Figure 2B). The binding affinity of PC1 suggests that the guanine riboswitch can tolerate modifications on the ligand pyrimidine ring that are not strongly deleterious for complex formation (∼100 nM vs ∼5 nM for PC1 and guanine, respectively). The binding affinity of PC1 is very similar to that of hypoxanthine, which is a naturally occurring guanine analog [25].

To explore the antibacterial activity spectrum of PC1, we used several Gram-positive bacterial species which are potential human pathogens containing guanine riboswitches. Of the 15 species tested, 9 showed marked cellular growth inhibition, including MDR strains and the *C. difficile* CD6 isolate representing the hypervirulent NAP1/027 strain (Figure 3B). Interestingly, when analyzing guanine riboswitch-regulated genes, we observed that all PC1-responsive strains had *guaA* under riboswitch control whereas the PC1-unresponsive ones did not employ riboswitch regulation to control *guaA*. The best example of this correlation is that while 16S rDNA sequence analysis indicates that *B. subtilis* and *Bacillus halodurans* are very closely related species [29], *B. halodurans* has a *guaA*-controlled riboswitch and is sensitive to PC1 whereas *B. subtilis* lacks a *guaA-*controlled riboswitch and is resistant to PC1. Antibiogram results also showed that strains exhibiting pronounced MDR phenotypes are sensitive to PC1 suggesting that the antimicrobial activity does not involve action mechanisms common to other known antibiotics.

Because our data suggest that PC1 acts by repressing the GMP synthetase *guaA*, we reasoned that the PC1 inhibitory activity should be reduced by GMP supplementation. *S. aureus* cells were thus grown with or without supplemented GMP, and colony forming units (CFU) were determined following serial microdilutions (Figure 3C). As predicted, bacterial growth inhibition was relieved when GMP was provided to cells grown in presence of PC1 for 2 h or 4 h, supporting the hypothesis that bacterial growth inhibition is caused by the riboswitch-mediated *guaA* gene repression that results in GMP cellular depletion.

The PC1 specificity was also confirmed using the Gram-negative bacterium *Escherichia coli* ATCC 35695, a strain that does not contain guanine riboswitches. As expected, *E. coli* showed no growth inhibition in presence of PC1 even when using strains deficient for the AcrAB efflux system or having increased membrane permeability (Figure S4). These results suggest that the inability of PC1 to inhibit *E. coli* most probably results from *guaA* not being under the control of a guanine riboswitch in *E. coli*.

### Bactericidal activity and specificity of PC1

**Figure 4:**
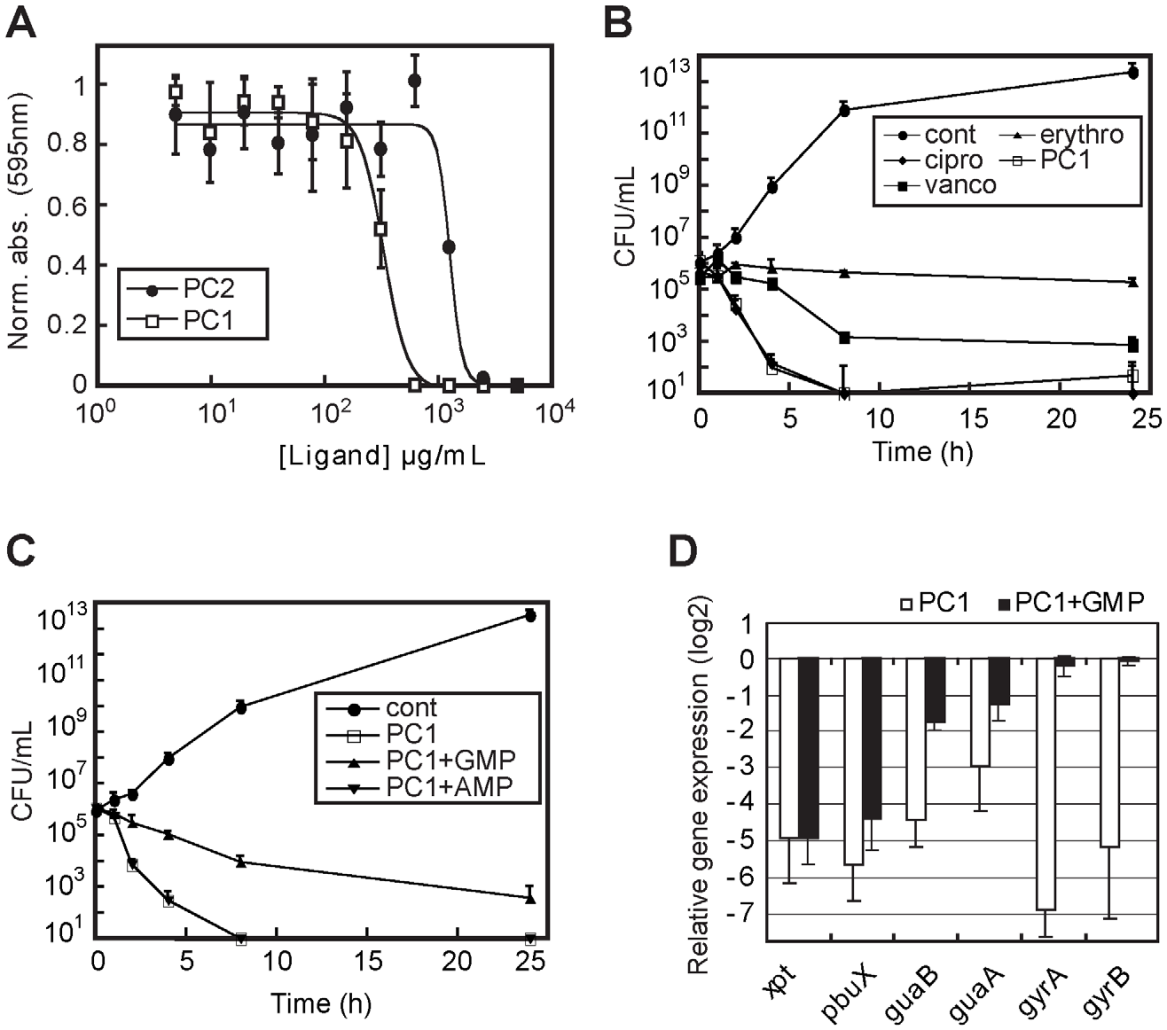
PC1 shows bactericidal activity through cellular GMP depletion. (A) Minimal inhibitory concentrations (MIC) determination of PC1 and PC2 on *S. aureus* strain ATCC 29213 in CAMHB. MIC values of 600 µg/mL and 5000 µg/mL were obtained for PC1 and PC2, respectively. (B) Bactericidal activity of PC1 and other known antibiotics against *S. aureus* as a function of time. For determination of the bactericidal effect of PC1, bacteria were inoculated at 10^5^ CFU/mL in absence (cont) or presence of 0.5 µg/mL ciprofloxacin (cipro), 0.5 µg/mL erythromycin (erythro), 1 µg/mL vancomycin (vanco) and 600 µg/mL PC1. The concentration of each antibiotic corresponds to their MIC. (C) Bactericidal activity of PC1 against *S. aureus* as a function of time in absence (cont) or presence of 600 µg/mL PC1, and in presence of PC1 with 100 µM GMP or AMP. (D) Relative expression of *S. aureus* genes under the control of a guanine riboswitch when grown in presence of PC1 or PC1 with GMP. Results obtained in presence of PC1 are normalized using *xpt* gene expression. Bacteria were inoculated at 10^8^ CFU/mL in CAMHB in absence or presence of 600 µg/mL PC1 with or without 100 µM GMP. Each experiment was performed three times and the average as well as standard deviations are shown.

To further characterize the riboswitch inhibitory action mechanism of PC1, *S. aureus* cells were grown in CAMHB in presence of various ligand concentrations. We obtained a PC1 dose-dependent growth inhibition response characterized by a MIC of 0.625 mg/mL (Figure 4A). PC2 was also used and its antibiotic activity was found to be less efficient than PC1, as observed in *B. subtilis* (Figure S2). When compared to known antibiotics, PC1 was found to have an extremely rapid bactericidal activity similar to ciprofloxacin, one of the most bactericidal antibiotics (Figure 4B). For instance, a 4 h treatment with PC1 led to 6.67±0.58 and 5.42±1.02 log reductions in CFU/mL compared to the untreated control for cultures of *S. aureus* ATCC 29213 and *C. difficile* CD6, respectively. When the same experiment was repeated by adding either GMP or AMP to the culture for 8 hours, bacterial growth was restored by a factor of 10^3^ only in presence of GMP (Figure 4C), suggesting that PC1 growth inhibition activity is specific to guanine metabolism.

To analyze the PC1-mediated riboswitch inhibition on *S. aureus* gene expression, we performed a transcriptomic microarray analysis containing a selection of *S. aureus* genes involved in different cellular processes such as virulence, secretion, general stress responses, sensory/regulatory systems, antibiotic resistance, iron transport and general biosynthesis [30] (Figure 4D). Among the 468 genes analyzed, 72% were repressed by at least two folds when *S. aureus* was treated with PC1 where the 16S rRNA gene was the most repressed (Table S1). This result is consistent with a riboswitch-mediated *guaA* gene expression inhibition leading to GMP cellular depletion and RNA synthesis inhibition. This is supported by the low expression of the guanine riboswitch operon (*xpt*, *pbuX*, *guaB* and *guaA*) as well as the two DNA gyrase subunits (*gyrA* and *gyrB*), which were used as housekeeping gene controls (Figure 4D). Of all the monitored genes in the microarray analysis, only *ahpF* and *ahpC*, two genes involved in stress response mechanisms, were activated by the PC1 treatment. However, when *S. aureus* was treated with PC1 and GMP, the microarray data showed an expression profile in which only 21% of the genes surveyed were repressed. Whereas the housekeeping gyrase genes were no longer repressed, the expression of the guanine riboswitch operon was still reduced, consistent with PC1 binding the riboswitch operon and inhibiting gene expression. The other repressed genes mainly comprised those involved in virulence and cell wall synthesis suggesting that the GMP supplemented cells were still under stress [31], which is in agreement with the partial growth rescue observed in Figure 4C. GMP is able to rescue PC1-treated cells in a dose-dependent manner (data not shown) but its low solubility prevents full recovery at higher doses. It is also probable that GMP-related feedback inhibitory mechanisms were responsible for some of the gene repression observed (as in the case of the guanylate kinase *gmk*). Taken together, these results are consistent with PC1 mainly acting through a riboswitch inhibition mechanism that ultimately results in GMP cellular deprivation and *S. aureus* growth inhibition.

### PC1 inhibits *S. aureus* growth in a murine model

**Figure 5:**
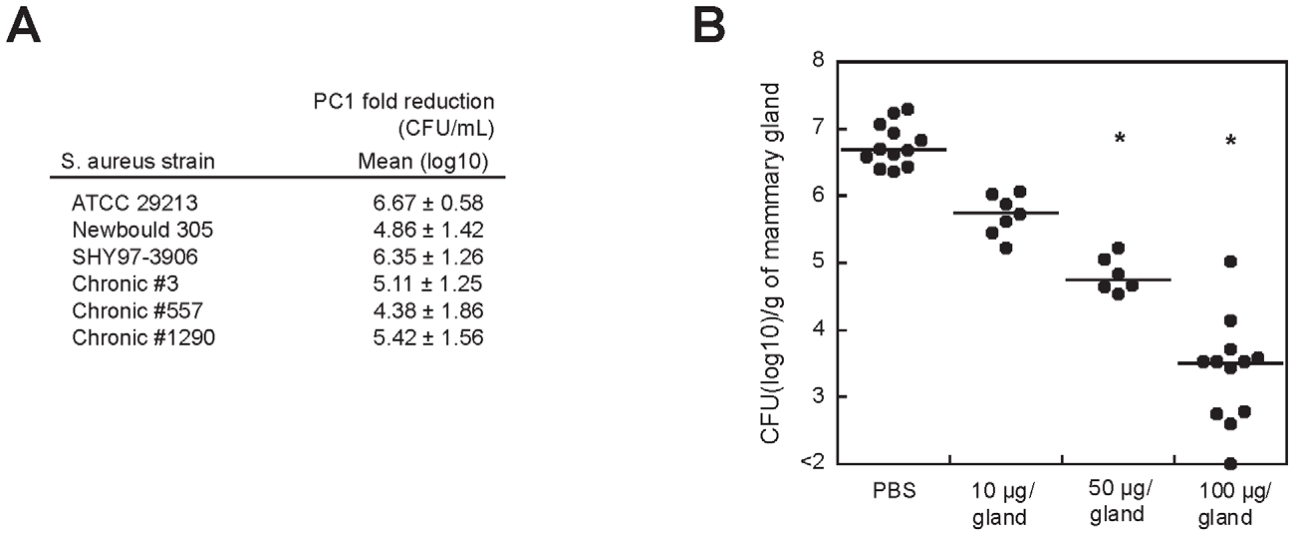
PC1 inhibits *S. aureus* clinical isolates *in vitro* and *in vivo.* (A) Fold reduction in viable counts (log10 CFU/mL) for the reference *S. aureus* strain ATCC 29213 and for selected bovine isolates after a 4 h exposure to PC1 as compared to the untreated culture. Newbould 305 (ATCC 29740) and SHY97-3906 are bovine isolates from typical mastitis cases and isolates 3, 557 and 1290 were from cows with persisting intra-mammary infections and chronic mastitis. (B) Bacterial counts (CFU) obtained from mice mammary glands 10 h post-infection with *S. aureus*. Mice mammary glands were treated (intra-mammary administration) 4 h after infection with PBS with or without PC1 at 10, 50 or 100 µg/gland. Each dot represents the CFU of each individual gland (n = 6–12) and the median value for each group is indicated by the bar. Statistical differences (P<0.05) between CFU recovered from treated and untreated animals are shown by asterisks (non-parametric Kruskal-Wallis ANOVA with Dunn's post test).

Because our data showed that the growth repression activity of PC1 is influenced by the presence of GMP, we decided to assess the bactericidal activity of PC1 in a murine mastitis model of *S. aureus* infection, which adequately represents the clinical context. Indeed, in addition to morbid nosocomial infections caused by *S. aureus*, this bacterium is one of the major pathogen leading to bovine mastitis, which is the most frequent and costly disease for dairy producers with current antibiotic therapies usually failing to eliminate infections from dairy herds [32]. The antimicrobial activity of PC1 was therefore first tested on several *S. aureus* isolates from mastitic cows, some of which having persisting chronic infections (Figure 5A). A bactericidal effect of at least 4 orders of magnitude was observed after a 4 h treatment with PC1. Next, to ascertain that *guaA* was expressed *in vivo* and that this gene may be important during infection, we monitored the expression level of *guaA* by real-time PCR. When strain 1290 was grown either in broth culture *in vitro* or when it was directly isolated from the mastitic milk of infected cows (*M. Allard and F. Malouin, in preparation*), very similar expression levels were found for *guaA* and the essential gene *gyrB* in both environments. This suggests that PC1 could have an impact on *guaA* expression *in vivo* and thus be used to treat *S. aureus* infections.

The proof of concept for the therapeutic efficacy of PC1 was established in our murine model of *S. aureus-*induced mastitis [33]. At 4 h post-infection, different concentrations of PC1 were administered to infected mice that were sacrificed 6 h later (Figure 5B). When compared to mice that were not treated with PC1, viable bacterial counts in the mammary gland were drastically reduced in a dose-dependent manner. This strong therapeutic effect was highly comparable to what we observed with known antibiotics. For example, amoxicillin decreased the bacterial load in the mammary gland to a log10 median value of 3.97 CFU/g of gland at a dose of 50 µg/gland. Noteworthy, a dose of 50 µg of amoxicillin would represent 100xMIC/g of gland, whereas a similar dose would only represent a twelfth of the MIC/g of gland for PC1. This result is consistent with the idea that PC1 is most efficient in the mammary gland environment suggesting that the microaerobic condition (i.e., low oxidative environment) of the mastitic milk[34] helps PC1 therapeutic efficacy. Consistent with these results, we found that the potency of PC1 was significantly increased by preventing its oxidation using a reductive agent such as DTT in susceptibility tests *in vitro* (Figure S5).

## Discussion

Despite previous large scale screen data suggesting that *guaA* is not essential for *S. aureus* growth [23], [35] in relatively rich media, we show here that blocking *guaA* expression can lead to bactericidal activity in various bacterial species. In support of *guaA* for cell viability, it has been recently reported that mutations occurring in *guaA* prevent *Streptococcus suis* [36] and *Salmonella thyphimurium* [37] from properly infecting porcine and murine models, respectively, suggesting that GMP bioavailability may be reduced during host infection and that *guaA* is likely to be crucial for bacterial infection in mammals. Thus, together with studies showing the importance of *guaA* for bacterial growth in urine or blood [38], [39], our data suggest that mammalian infection sites may significantly differ in their nutrient compositions from those used in large scale screens [23], [35], and that care should be taken when assessing the “essentiality” of a gene. Furthermore, when assessing whether *S. aureus* could develop resistance to PC1, no resistant bacteria were obtained after more than 30 passages suggesting that maintaining a functional *guaA-*regulated riboswitch is a vital process (Figure S6). Taken together, the demonstration that *guaA* expression is normally maintained in *S. aureus* grown *in vivo* and the strong therapeutic effect resulting from PC1 treatment indicate that *guaA* is an important contributor to the survival of *S. aureus* during infection and that it can be used as an antibiotic target.

**Figure 6:**
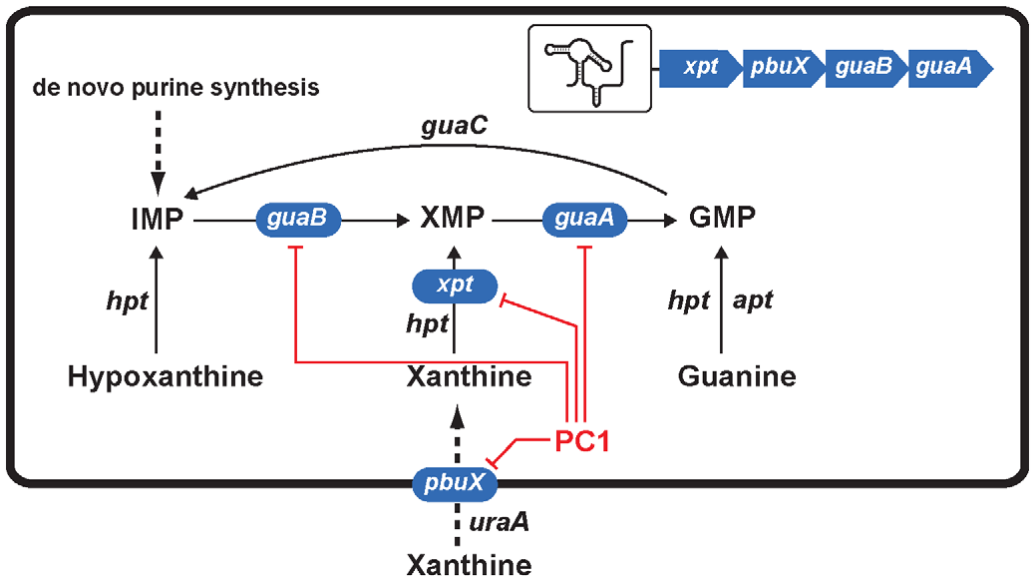
Scheme representing the action mechanism of PC1 on *S. aureus* guanine pathway. Genes highlighted in blue correspond to those of the operon *xpt-pbuX-guaB-guaA* that is controlled by the guanine riboswitch. Red lines indicate genes that are inhibited by PC1 via its binding to the guanine riboswitch. Of these four inhibited genes, *guaA* and *guaB* are known to be critical for guanine nucleotide biosynthesis [22], [23], [36], [37], [38], [39] and their inhibition very likely lead to the repression of GMP synthesis and most probably of RNA and DNA production.

The major limitation to validate an antibiotic that targets riboswitches is to evaluate the antibiotic specificity of action. In this particular case, PC1 is not a broad-spectrum antibacterial drug given that it does not target all bacteria containing guanine riboswitches, but only those in which *guaA* is under the control of a riboswitch. It is not excluded that other riboswitch-controlled genes may participate in the PC1-dependent bacterial growth inhibition (e.g., *guaB*), or that PC1 may bind other cellular targets, which alone or in combination with the riboswitch-controlled gene repression, would repress bacterial growth. Nevertheless, the restricted nature of growth inhibition likely indicates that PC1 inhibits bacterial growth through riboswitch binding and not via an alternative mechanism such as DNA incorporation (Figure 6). For instance, when performing antibiograms using the guanine analog 6-thioguanine, a general growth inhibition was observed in *E. coli* and *S. aureus* (Figure S7), consistent with its incorporation into DNA that perturbs the epigenetic pathway of gene regulation [40]. The selective antibacterial activity of PC1 toward *S. aureus* was also supported by the lack of apparent toxicity for mice treated with the experimental compound at concentrations as high as 100 µg/gland with no sign of discomfort including vocalizations, curved back, piloerection and hypothermia. There was also no apparent cytotoxicity upon histological observations of mammary tissues in PC1-treated mice compared to PBS-treated glands (Figure S8).

Recently, the Breaker group used purine derivatives modified in position 2 or 4 to target guanine riboswitches and found three molecules that inhibited bacterial growth [15]. Among these molecules, only one was able to repress the expression of a guanine riboswitch-controlled reporter gene suggesting that at least two molecules inhibited cell growth through a different mechanism of action. It is possible that the mode of action of these two molecules involves nucleic acids incorporation following ribosylation at position 9 of the purine analog, as demonstrated for 6-thioguanine. It is also interesting to mention that the antibiotic activity was only observed using *B. subtilis* strains cultivated in minimal medium whereas no growth inhibition was detected in rich media. In our study, we selected guanine riboswitch ligands that cannot be ribosylated to prevent alternative modes of action, and favored a pyrimidine compound (PC1) that retained most of the key functional groups. By testing various bacterial species, we observed that the bactericidal activity of PC1 was only seen against bacteria using a guanine riboswitch to control *guaA* expression, and this even when bacteria were grown in a rich medium. This demonstrates that analog binding to riboswitch aptamers is not the only determinant to achieve selective and efficient bactericidal effects.

This study shows for the first time that antibiotics targeting riboswitches may be efficient to kill bacterial pathogens *in vitro* as well as in mammalian infection models. We found here that the selective bacterial killing of PC1 is only achieved when *guaA*, a GMP synthetase, is under the control of the riboswitch and when the antimicrobial agent cannot be ribosylated. The narrow spectrum of activity we demonstrated here for PC1 is very interesting since two of the target bacteria, *S. aureus* and *C. difficile*, are among the most problematic nosocomial pathogens. The spread of MDR in those bacterial species also stresses the importance to develop new antibiotics that avoid current mechanisms of resistance. The use of narrow spectrum drugs should be encouraged whenever possible to reduce any selective pressure for resistance in non-targeted bacteria. Here, we also showed that the development of resistance toward PC1 is likely to be infrequent.

## Methods

### Ethics statement

The institutional ethics committee on animal experimentation of the Faculté des sciences of the Université de Sherbrooke (QC, Canada) approved these experiments and the guidelines of the Canadian Council on Animal Care were respected during all the procedures.

### Reagents

4-hydroxy-2,5,6-triaminopyrimidine (PC1), 2,4-diamino-6-hydroxypirimidine (PC2) and 9-methylguanine were purchased from Fluka. 2-amino-5-bromo-6-methyl-4-pyrimidol and 2-amino-4-hydroxy-6-methylpyrimidine were purchased from Aldrich.

### Strategy of ligand selection

Our ligand selection took into account guanine binding requirements [25] and crystal structure interactions [21], [26], [27]. Planar molecules were selected to preserve stacking interactions with adenines 21 and 52 in the guanine aptamer binding site. One of our important selection criteria was to avoid the presence of functional groups that could serve as a ribosylation site in purine or pyrimidine analogs that would allow subsequent nucleic acid incorporation and non-specific antibiotic effect. Successfully identified molecules were drawn using Chem3D Pro (CambridgeSoft) and docked onto the guanine aptamer crystal structure (PDB 1U8D). The *in silico* procedure was important to validate aptamer-ligand interactions and to avoid sterical obstructions that would perturb ligand binding.

### Transcription of RNA

For the production of guanine riboswitch aptamers, DNA templates were prepared from partial duplexes and transcribed using T7 RNA polymerase as previously described [28]. The aptamer sequences used in this study are based on the genomic sequence to which a GCG sequence is added to the 5′ side to allow high transcription yield and to minimize the 5′ heterogeneity [28].

### In-line probing assays

[5′-^32^P] RNA molecules were incubated for 96 h at 25°C in 50 mM Tris-HCl buffer, pH 8.5, 20 mM MgCl_2_ and 100 mM KCl in absence or in presence of indicated ligand concentrations. The reactions were stopped with a 97% formamide solution containing 10 mM EDTA and samples were purified by electrophoresis in 10% polyacrylamide gels (acrylamide:bisacrylamide; 19:1) containing 8 M urea. Gel were dried and exposed to Phosphor Imager screens.

### βeta-galactosidase assays

Regulation of the beta-galactosidase reporter gene expression in presence of PC1 or PC2 was determined using an *xpt-lacZ* transcriptional fusion construct integrated in the genome of *B. subtilis* by recombination. The beta-galactosidase activity was measured after 4 h of growth at 37°C in minimal medium in absence or presence of the indicated ligand concentrations [41].

### Antibiogram assays

Bacteria were inoculated at 10^5^ CFU/mL in melted Muller-Hinton agar. After agar medium was solidified, six wells of 4 mm in diameter were made and filled with 10 µL of the tested molecules (5 mg/mL). Plates were incubated for 16 h at 37°C.

### Antibiotic minimal inhibitory concentrations

The minimal inhibitory concentration (MIC) of PC1 and PC2 against *S. aureus* strain ATCC 29213 was determined using a microdilution method in 96-well plates [30]. Bacteria were inoculated at 10^5^ CFU/mL and incubated at 37°C for 24 h in cation-adjusted Muller-Hinton broth (CAMHB). Bacterial growth was detected by measuring the OD at 595 nm on a microplate reader.

### Antibiotic bactericidal activity

Time-kill experiments were performed for the determination of the bactericidal effect of test antibiotics. Bacteria were inoculated at 10^5^ CFU/mL in CAMHB in absence or presence of the antibiotic at its MIC with or without 100 µM GMP. Bacterial permeability to GMP was increased by adding 0.002% Triton X-100. At several time points, bacteria were sampled and serially diluted before spreading on tryptic soya agar (TSA) plates for CFU determinations. Plates were incubated for 24 h at 37°C.

### Transcriptomic microarray

Bacteria were inoculated at 10^8^ CFU/mL in CAMHB in absence or presence of 600 µg/mL PC1 or 600 µg/mL PC1 supplemented with 100 µM GMP. After 30 min of growth, RNA was extracted and 2.5 µg of RNA were submitted to reverse transcription to generate fluorescent probes through an aminoallyl cDNA labeling procedure before being hybridized on the microarray [30].

### Murine mastitis model

Experimental conditions used here for the mastitis model were previously optimized for *S. aureus* Newbould and antibiotic treatment [42]. CD-1 lactating mice (Charles River, St. Constant, Canada) were used 12 to 14 days after offspring birth and typically weighed 35 to 40 g. Pups were removed 1 h before bacterial inoculation of mammary glands and a mixture of ketamine/xylazine at 87 and 13 mg/kg of weight, respectively, was used for anesthesia of lactating mice. A 100 µl syringe with a 33-gauge blunt needle was used to inoculate both L4 (on the left) and R4 (on the right) abdominal mammary glands. These large glands constitute the fourth pair found from head to tail. Each udder canal was exposed by a small cut at the near end of the teat under a binocular and 100 µL of bacterial suspension (1 CFU/µL) was injected through the orifice. Mice mammary glands were treated 4 h after infection with PBS or PBS with 10, 50 and 100 µg/gland of PC1 and mice were sacrified 6 h later for mammary gland sampling and homogenization. The tissues used for CFU counts were homogenized in 2 mL of PBS and the bacterial content was evaluated by serial logarithmic dilutions on agar. The detection limit was 100 CFU/g of gland.

## Supporting Information

Figure S1 Guanine and guanine-related metabolic pathways in different bacterial species. Guanine riboswitch-regulated genes are shown using a color scheme where *Bacillus subtilis, Clostridium difficile* and *Staphylococcus aureus* are indicated in green, orange and blue, respectively. Dashed lines represent metabolite membrane transporters. The synthesis of GMP is highly dependent upon *guaA* and *guaB*, which respectively encodes a GMP synthetase and an IMP dehydrogenase. While *guaB* has been shown to be essential in *B. subtilis* [23], mutations in *guaA* have been reported to make cells auxotroph for guanine [22]. The importance of *guaA* and *guaB* has also been observed during bacterial infections in murine and porcine models [36], [37]. Thus, *guaA* and *guaB* encode two enzymes critical for guanine nucleotide biosynthesis and reduction of their expression via riboswitch action is very likely to produce bacterial growth inhibition.
(0.32 MB TIF)

Figure S2 *B. subtilis* growth is inhibited by PC1 acting as a guanine riboswitch antibiotic. (A) Minimal inhibitory concentrations (MIC) of PC1 and PC2 against *B. subtilis*. The MICs were determined using a microdilution method in 96-well plates. Bacteria were inoculated at 10^5^ CFU/mL in cation-adjusted Muller-Hinton broth (CAMHB) or in minimal media (MM) and incubated at 37°C for 24 h. MIC values of 1250 µg/mL and 5000 µg/mL were respectively obtained for PC1 and PC2 in MM, but no growth inhibition was observed in CAMHB. (B) PC1 modulates riboswitch activity in CAMHB in a dose-dependent manner. Beta-galactosidase activity of a *xpt* riboswitch-*lacZ* transcriptional fusion construct integrated by recombination in the genome of *B. subtilis* was assayed after 4 h of growth at 37°C in CAMHB in the absence or presence of the indicated ligand concentrations. This confirms that PC1 can modulate riboswitch activity in a relatively rich media such as CAMHB and not only in a minimal media (Figure 2D), where growth inhibition is observed. Each experiment was performed three times and the average as well as the SD are shown.
(0.39 MB TIF)

Figure S3 Schematic representation of the *S. aureus xpt-pbuX-guaB-guaA* operon controlled by the guanine riboswitch. (A) The predicted number of nucleotides for each intergenic region is shown. (B) RT-PCR of intergenic regions performed using total RNA. Note that an amplification product is obtained in all cases indicating that *xpt, pbuX, guaB* and *guaA* are included in an operon controlled by the guanine riboswitch. RNA was extracted from lysate using a Quiagen RNeasy kit and treated with DNase I in presence of RNase inhibitors. Following this, 1 µg was used for reverse transcription with 200 units of SuperScript II (Invitrogen), using 100 pmoles of a DNA oligonucleotide used as a primer. The reaction was performed at 42°C for 1 h and used in a PCR reaction using an appropriate forward primer. Lane L represents a 100 bp ladder with the number of base pairs indicated for each band. Lanes *xpt-pbuX, pbuX-guaB* and *guaB-guaA* represent PCR reactions amplifying corresponding intergenic regions.
(1.02 MB TIF)

Figure S4 PC1 does not inhibit the growth of the Gram negative bacterium *E. coli*, which does not naturally contain guanine riboswitches. The MIC of PC1 and other known antibiotics against *E. coli* ATCC 35695 were determined using a broth microdilution method. As expected, PC1 does not show any antibiotic activity toward *E. coli* ATCC 35695, most probably because of the absence of a guanine riboswitch. However, to exclude the possibility that the lack of inhibitory activity is due to a poor cell penetration of PC1 into *E. coli* or to an active efflux of this compound out of the cells, we also tested PC1 activity against two isogenic *E. coli* mutants. While *E. coli* ATCC 35695 is a standard strain, AcrAB is deficient for the multidrug efflux pump AcrAB [43] and Imp has an increased membrane permeability [44]. Our results show that there is still no inhibitory activity of PC1 against any of the mutants. Please note that the Imp increased membrane permeability is confirmed by the antibiotic activity of vancomycin, which is a large glycopeptide molecule for which Gram negative bacteria are normally impermeable. Besides, the reduced efflux activity of AcrAB is verified from the ability of erythromycin to inhibit bacterial growth. Erythromycin is also able to inhibit Imp bacterial growth due to the increased membrane permeability. These results indicate that the lack of PC1 antibiotic activity toward *E. coli* is not due to its active efflux by the bacteria or its inability to pass through the cell membrane. All concentrations are in µg/mL and the chemical structure for PC1 is shown in Figure 2B.
(0.12 MB TIF)

Figure S5 DTT increases the antibiotic activity of PC1 probably by preventing its oxidative self-condensation. (A) MICs were determined using a microdilution method in 96-well microplates in absence or presence of DTT at the concentrations shown. Bacteria were inoculated at 10^5^ CFU/mL and incubated at 37°C for 24 h in CAMHB. Please note that the MIC is decreased by a factor of ∼40x in the presence of 0.05% DTT. At low PC1 concentrations, it can be observed that DTT does not inhibit bacterial growth by itself. (B) Schematic representation of the probable oxidative self-condensation of PC1 that could be prevented by DTT. Previous studies have shown that 4,5-diaminopyrimidines can produce insoluble and deeply-colored orange substances such as pyrimido[5,4-g]- and pyrimido[4,5-g]pteridines by oxidative self-condensation [45]. Given that PC1 is structurally very similar to 4,5-diaminopyrimidines and that it produces an orange precipitate over time, it is likely that PC1 also self-condenses by air oxidation. This is consistent with the observation that a reductive agent such as DTT can slow down the formation of the precipitate (data not shown).
(0.37 MB TIF)

Figure S6 Serial passages in the presence of sub-inhibitory concentrations of test antibiotics demonstrating the inability of *S. aureus* to develop resistance toward PC1. The MIC of test compounds against *S. aureus* strain ATCC 29213 recovered from broth cultures containing sub-inhibitory concentrations of antibiotics (0.25X of MIC) was determined every 5 passages for up to 30 passages. As a comparison, results obtained with two known antibiotics, ciprofloxacin and rifampicin, were added to the histogram. High level resistance to ciprofloxacin and rifampicin is rapidly selected (within 5 daily passages) in *S. aureus*. Such rapid development of resistance for the traditional drugs is consistent with the selection of known single point mutations each able to provide a decrease in drug affinity for the bacterial cell target. There are at least 2 known point mutations in GyrA conferring resistance to ciprofloxacin in addition to possible over-expression of the NorA efflux pump system also occurring through mutations (at least 3 possible mutations) [46] and at least 17 possible different mutations in RpoB enabling resistance to rifampicin have been documented [47]. The absence of resistance observed in presence of PC1 is probably because reestablishing *guaA* gene expression in the presence of PC1 requires multiple mutational steps thus reducing the frequency of resistance development and/or that maintaining a functional riboswitch is a vital process that does not allow bacteria to bypass PC1 antibiotic action. PC1 experiments were performed three times and the average as well as the SD are shown.
(0.46 MB TIF)

Figure S7 Antibiograms performed on strains of *E. coli* ATCC 35695 and Methicilin Resistant *S. aureus* strain COL. *E. coli* ATCC 35695 (A) and *S. aureus* strain COL (B) were grown in absence (well #1) or in presence of 7.5 µg (well #2) or 15 µg 6-thioguanine (well #3). Please note that 6-thioguanine is able to be ribosylated (C) and incorporated in DNA[40], which probably explains its riboswitch-independent antibiotic activity toward both *E. coli* and *S. aureus*.
(3.01 MB TIF)

Figure S8 Histology of mice mammary glands treated with PC1. Mice were either injected with (A) 100 µL PBS or with (B) 100 µL PBS containing 100 µg of PC1. The treatment was allowed for 6 h and mammary glands were excised, fixed in 4% formaldehyde for 24 h at room temperature and embedded in paraffin wax. Hematoxylin-eosin staining was done on sections of 5 µm thickness. Magnifications on the pictures are 200×. The histology study reveals that there is no observable damage to the gland following PC1 injection.
(5.90 MB TIF)

Table S1 Transcriptomic microarray showing the relative expression of *S. aureus* genes as a function of PC1 and GMP.
(0.08 MB XLS)
