# Supplementary figures and images for: Novel Riboswitch Ligand Analogs as Selective Inhibitors of Guanine-Related Metabolic Pathways
Source: PLoS Pathog. 2010 Apr 22;6(4):e1000865. doi: 10.1371/journal.ppat.1000865 (PMC2858708; doi:10.1371/journal.ppat.1000865)

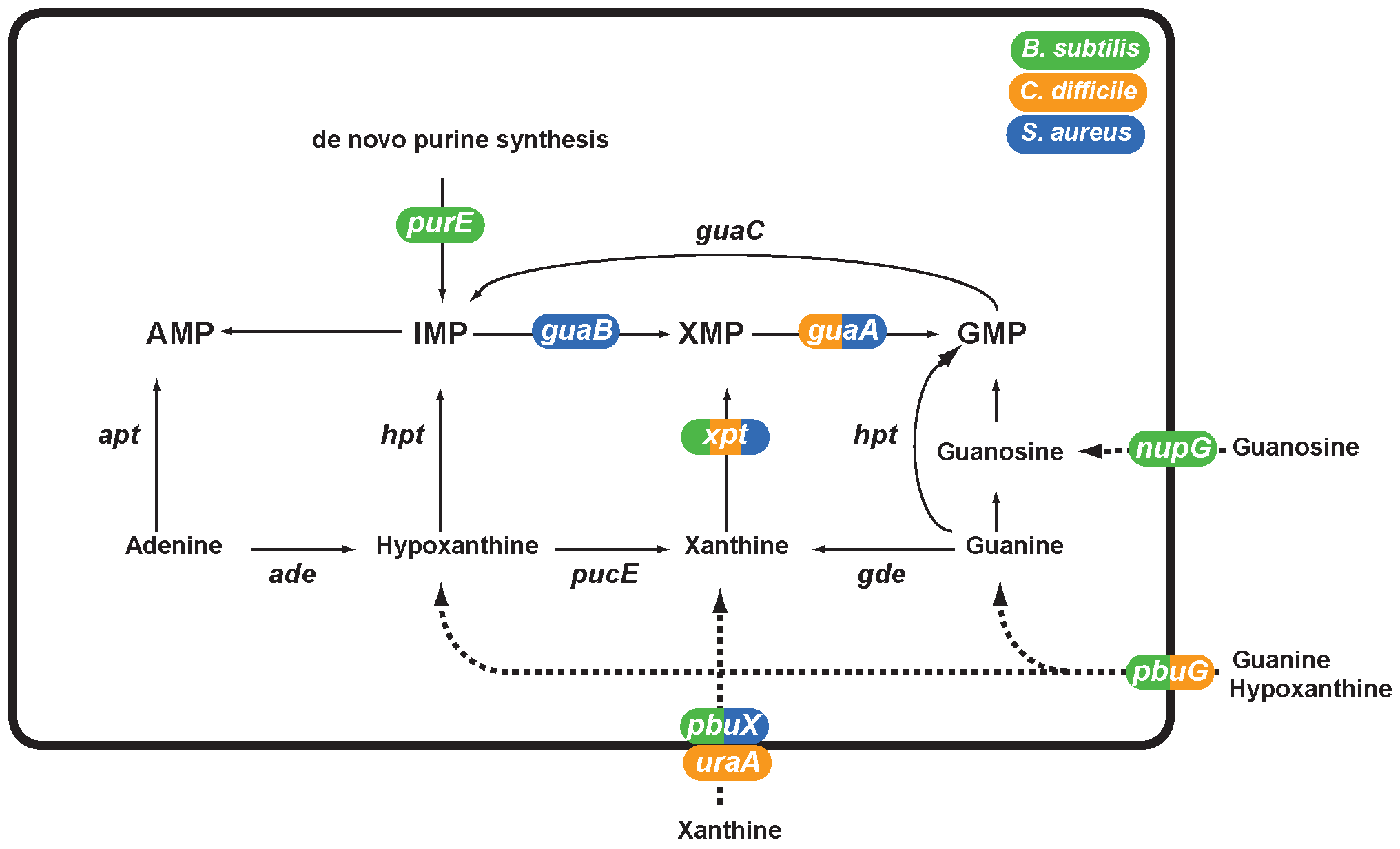

Supplement: Figure S1 — Guanine and guanine-related metabolic pathways in different bacterial species. Guanine riboswitch-regulated genes are shown using a color scheme where Bacillus subtilis, Clostridium difficile and Staphylococcus aureus are indicated in green, orange and blue, respectively. Dashed lines represent metabolite membrane transporters. The synthesis of GMP is highly dependent upon guaA and guaB, which respectively encodes a GMP synthetase and an IMP dehydrogenase. While guaB has been shown to be essential in B. subtilis [23], mutations in guaA have been reported to make cells auxotroph for guanine [22]. The importance of guaA and guaB has also been observed during bacterial infections in murine and porcine models [36], [37]. Thus, guaA and guaB encode two enzymes critical for guanine nucleotide biosynthesis and reduction of their expression via riboswitch action is very likely to produce bacterial growth inhibition. (0.32 MB TIF) [file ppat.1000865.s001.tif]

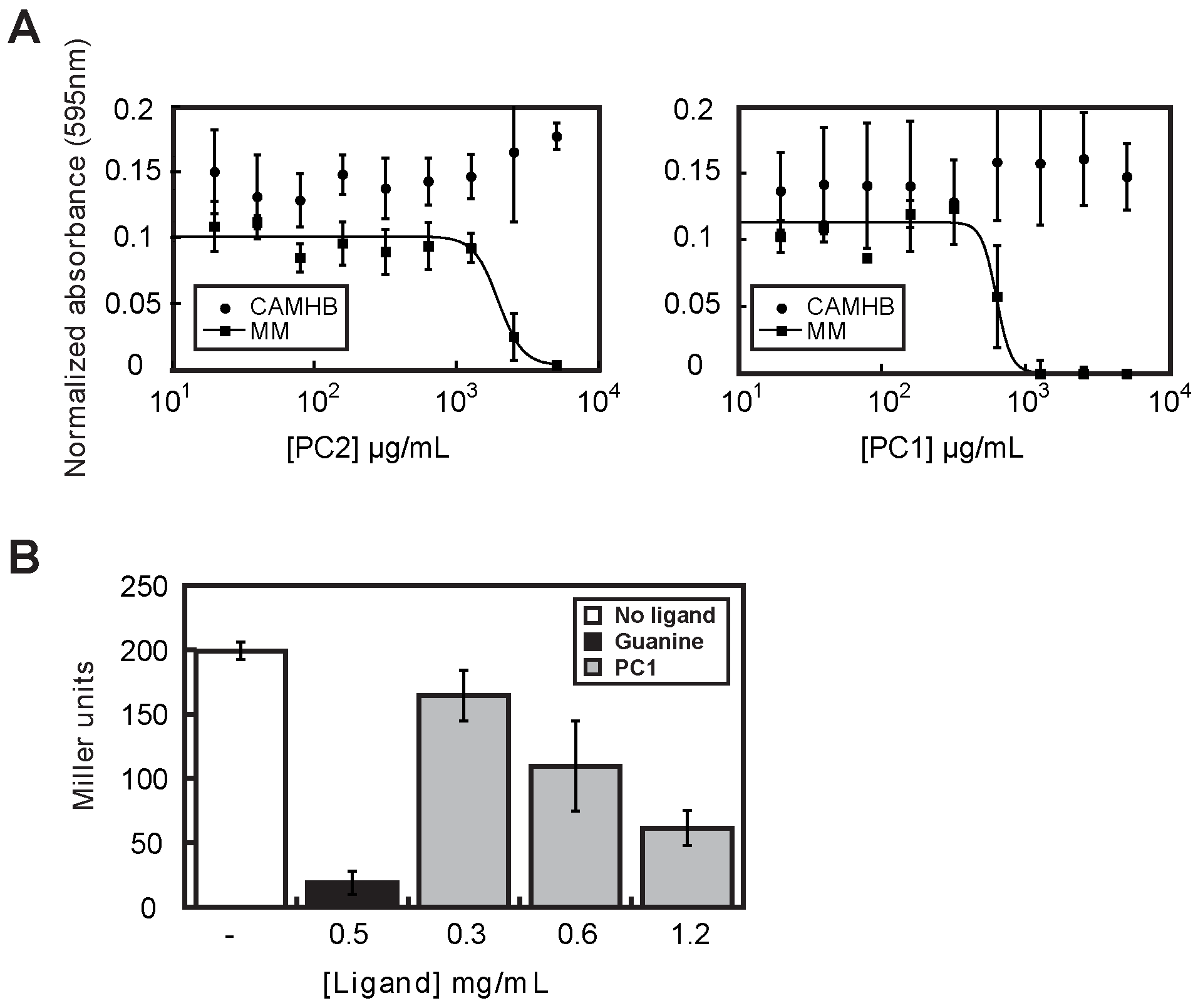

Supplement: Figure S2 — B. subtilis growth is inhibited by PC1 acting as a guanine riboswitch antibiotic. (A) Minimal inhibitory concentrations (MIC) of PC1 and PC2 against B. subtilis. The MICs were determined using a microdilution method in 96-well plates. Bacteria were inoculated at 105 CFU/mL in cation-adjusted Muller-Hinton broth (CAMHB) or in minimal media (MM) and incubated at 37°C for 24 h. MIC values of 1250 µg/mL and 5000 µg/mL were respectively obtained for PC1 and PC2 in MM, but no growth inhibition was observed in CAMHB. (B) PC1 modulates riboswitch activity in CAMHB in a dose-dependent manner. Beta-galactosidase activity of a xpt riboswitch-lacZ transcriptional fusion construct integrated by recombination in the genome of B. subtilis was assayed after 4 h of growth at 37°C in CAMHB in the absence or presence of the indicated ligand concentrations. This confirms that PC1 can modulate riboswitch activity in a relatively rich media such as CAMHB and not only in a minimal media (Figure 2D), where growth inhibition is observed. Each experiment was performed three times and the average as well as the SD are shown. (0.39 MB TIF) [file ppat.1000865.s002.tif]

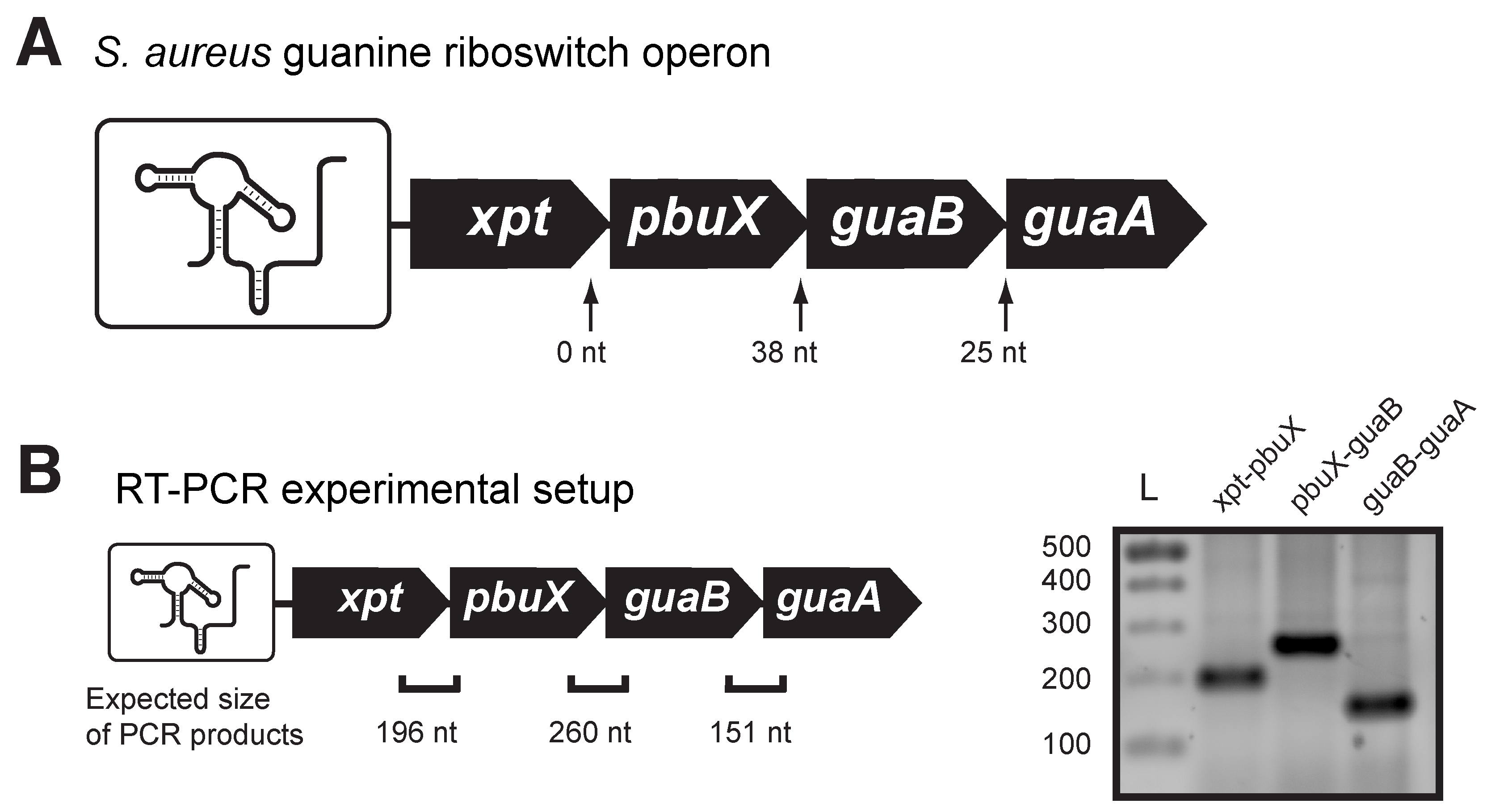

Supplement: Figure S3 — Schematic representation of the S. aureus xpt-pbuX-guaB-guaA operon controlled by the guanine riboswitch. (A) The predicted number of nucleotides for each intergenic region is shown. (B) RT-PCR of intergenic regions performed using total RNA. Note that an amplification product is obtained in all cases indicating that xpt, pbuX, guaB and guaA are included in an operon controlled by the guanine riboswitch. RNA was extracted from lysate using a Quiagen RNeasy kit and treated with DNase I in presence of RNase inhibitors. Following this, 1 µg was used for reverse transcription with 200 units of SuperScript II (Invitrogen), using 100 pmoles of a DNA oligonucleotide used as a primer. The reaction was performed at 42°C for 1 h and used in a PCR reaction using an appropriate forward primer. Lane L represents a 100 bp ladder with the number of base pairs indicated for each band. Lanes xpt-pbuX, pbuX-guaB and guaB-guaA represent PCR reactions amplifying corresponding intergenic regions. (1.02 MB TIF) [file ppat.1000865.s003.tif]

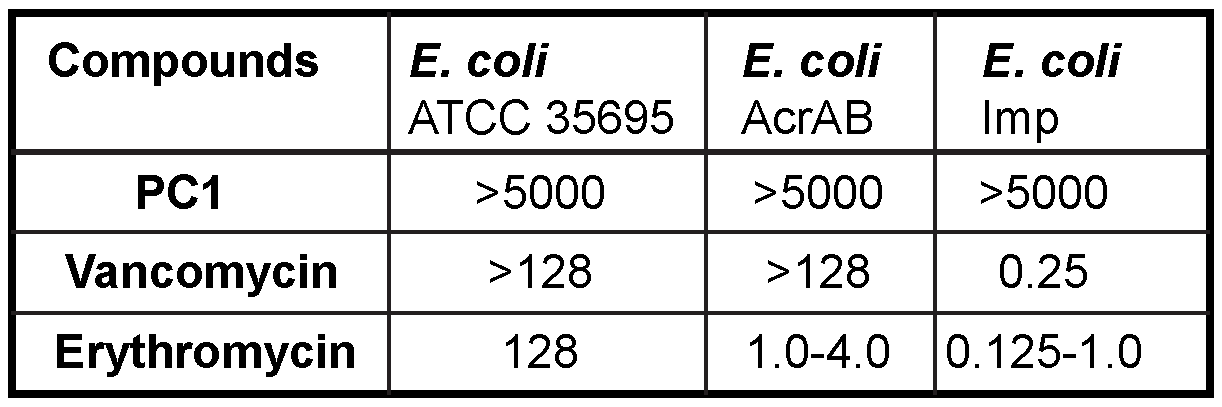

Supplement: Figure S4 — PC1 does not inhibit the growth of the Gram negative bacterium E. coli, which does not naturally contain guanine riboswitches. The MIC of PC1 and other known antibiotics against E. coli ATCC 35695 were determined using a broth microdilution method. As expected, PC1 does not show any antibiotic activity toward E. coli ATCC 35695, most probably because of the absence of a guanine riboswitch. However, to exclude the possibility that the lack of inhibitory activity is due to a poor cell penetration of PC1 into E. coli or to an active efflux of this compound out of the cells, we also tested PC1 activity against two isogenic E. coli mutants. While E. coli ATCC 35695 is a standard strain, AcrAB is deficient for the multidrug efflux pump AcrAB [43] and Imp has an increased membrane permeability [44]. Our results show that there is still no inhibitory activity of PC1 against any of the mutants. Please note that the Imp increased membrane permeability is confirmed by the antibiotic activity of vancomycin, which is a large glycopeptide molecule for which Gram negative bacteria are normally impermeable. Besides, the reduced efflux activity of AcrAB is verified from the ability of erythromycin to inhibit bacterial growth. Erythromycin is also able to inhibit Imp bacterial growth due to the increased membrane permeability. These results indicate that the lack of PC1 antibiotic activity toward E. coli is not due to its active efflux by the bacteria or its inability to pass through the cell membrane. All concentrations are in µg/mL and the chemical structure for PC1 is shown in Figure 2B. (0.12 MB TIF) [file ppat.1000865.s004.tif]

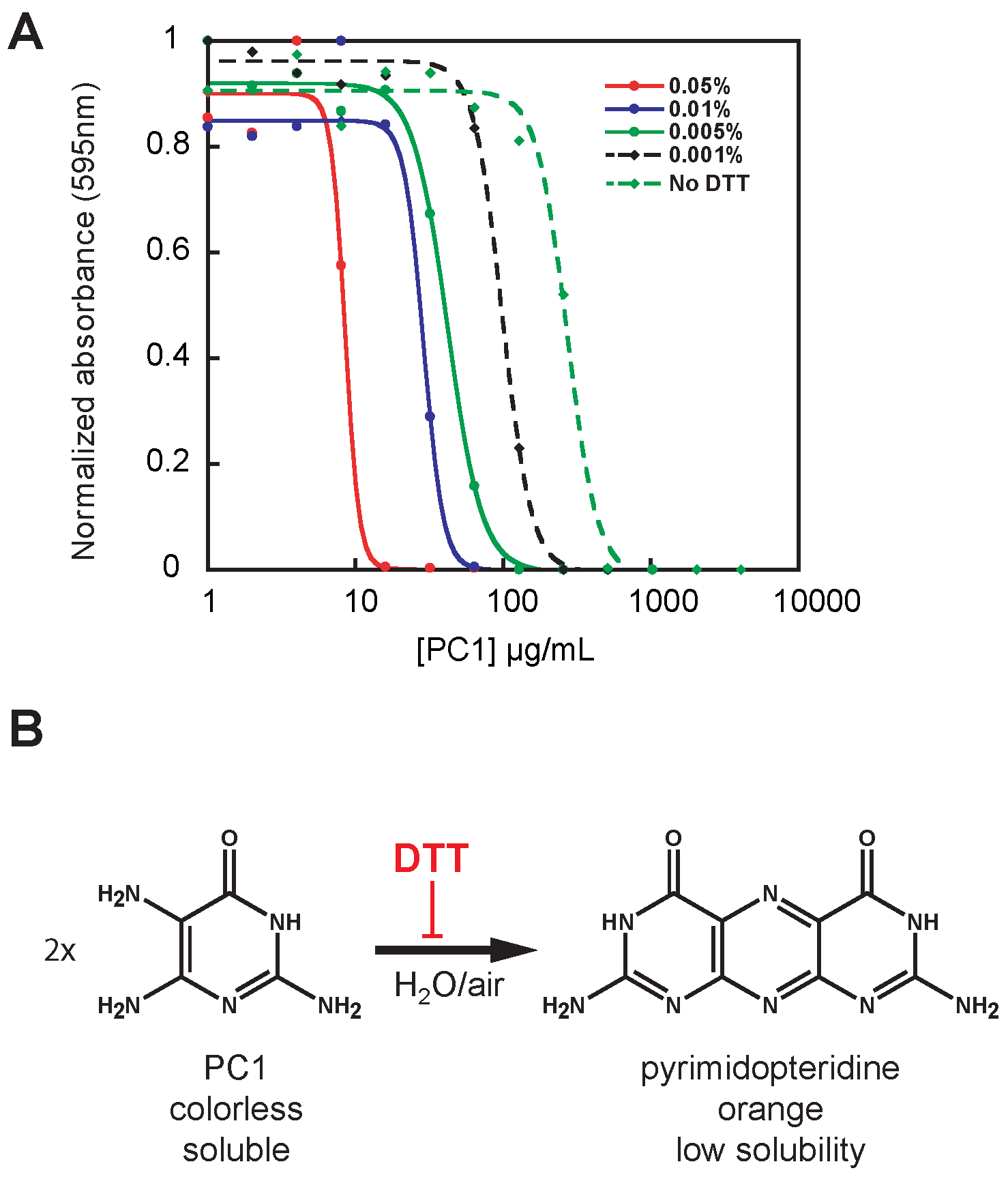

Supplement: Figure S5 — DTT increases the antibiotic activity of PC1 probably by preventing its oxidative self-condensation. (A) MICs were determined using a microdilution method in 96-well microplates in absence or presence of DTT at the concentrations shown. Bacteria were inoculated at 105 CFU/mL and incubated at 37°C for 24 h in CAMHB. Please note that the MIC is decreased by a factor of ∼40x in the presence of 0.05% DTT. At low PC1 concentrations, it can be observed that DTT does not inhibit bacterial growth by itself. (B) Schematic representation of the probable oxidative self-condensation of PC1 that could be prevented by DTT. Previous studies have shown that 4,5-diaminopyrimidines can produce insoluble and deeply-colored orange substances such as pyrimido[5,4-g]- and pyrimido[4,5-g]pteridines by oxidative self-condensation [45]. Given that PC1 is structurally very similar to 4,5-diaminopyrimidines and that it produces an orange precipitate over time, it is likely that PC1 also self-condenses by air oxidation. This is consistent with the observation that a reductive agent such as DTT can slow down the formation of the precipitate (data not shown). (0.37 MB TIF) [file ppat.1000865.s005.tif]

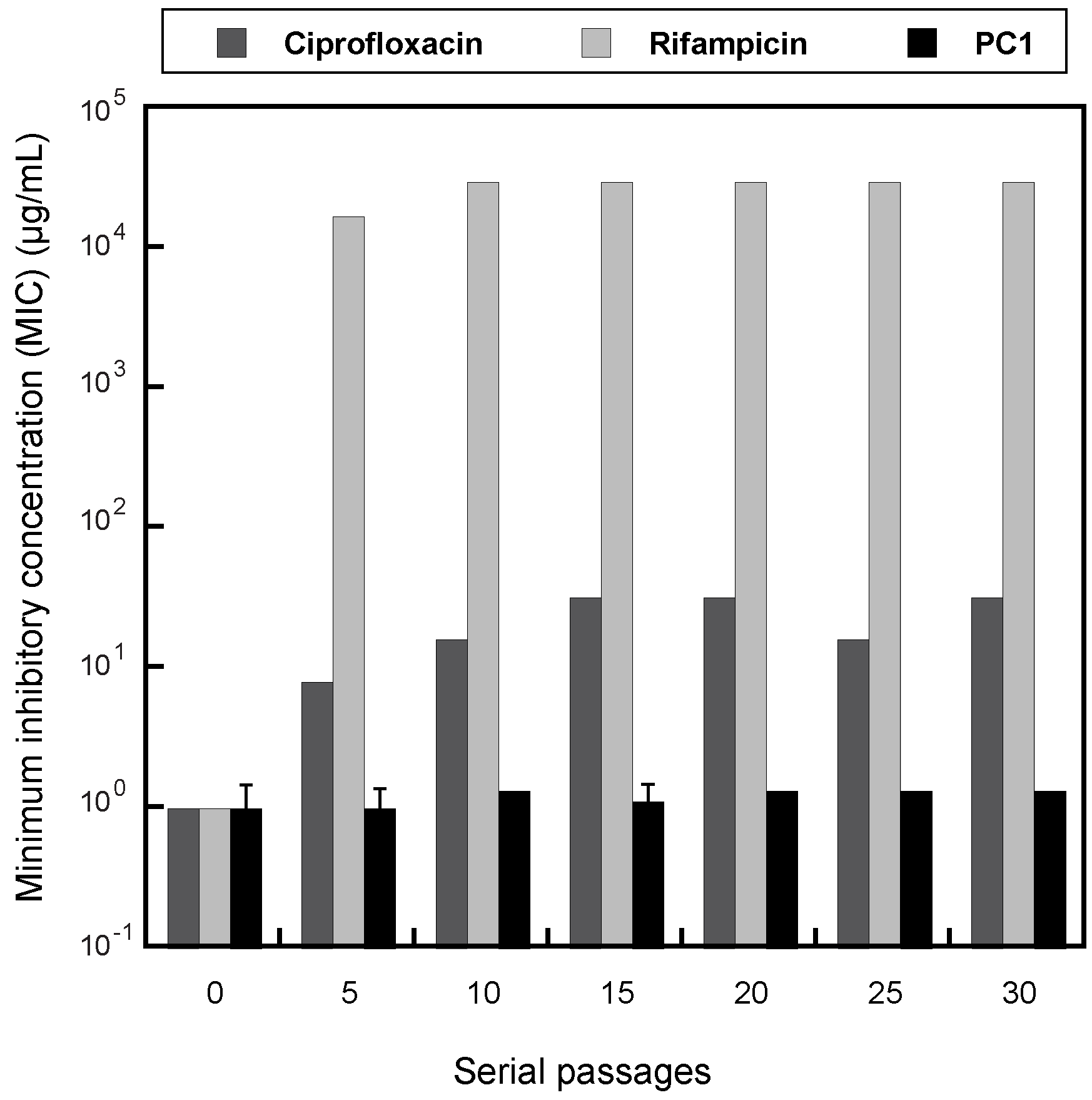

Supplement: Figure S6 — Serial passages in the presence of sub-inhibitory concentrations of test antibiotics demonstrating the inability of S. aureus to develop resistance toward PC1. The MIC of test compounds against S. aureus strain ATCC 29213 recovered from broth cultures containing sub-inhibitory concentrations of antibiotics (0.25X of MIC) was determined every 5 passages for up to 30 passages. As a comparison, results obtained with two known antibiotics, ciprofloxacin and rifampicin, were added to the histogram. High level resistance to ciprofloxacin and rifampicin is rapidly selected (within 5 daily passages) in S. aureus. Such rapid development of resistance for the traditional drugs is consistent with the selection of known single point mutations each able to provide a decrease in drug affinity for the bacterial cell target. There are at least 2 known point mutations in GyrA conferring resistance to ciprofloxacin in addition to possible over-expression of the NorA efflux pump system also occurring through mutations (at least 3 possible mutations) [46] and at least 17 possible different mutations in RpoB enabling resistance to rifampicin have been documented [47]. The absence of resistance observed in presence of PC1 is probably because reestablishing guaA gene expression in the presence of PC1 requires multiple mutational steps thus reducing the frequency of resistance development and/or that maintaining a functional riboswitch is a vital process that does not allow bacteria to bypass PC1 antibiotic action. PC1 experiments were performed three times and the average as well as the SD are shown. (0.46 MB TIF) [file ppat.1000865.s006.tif]

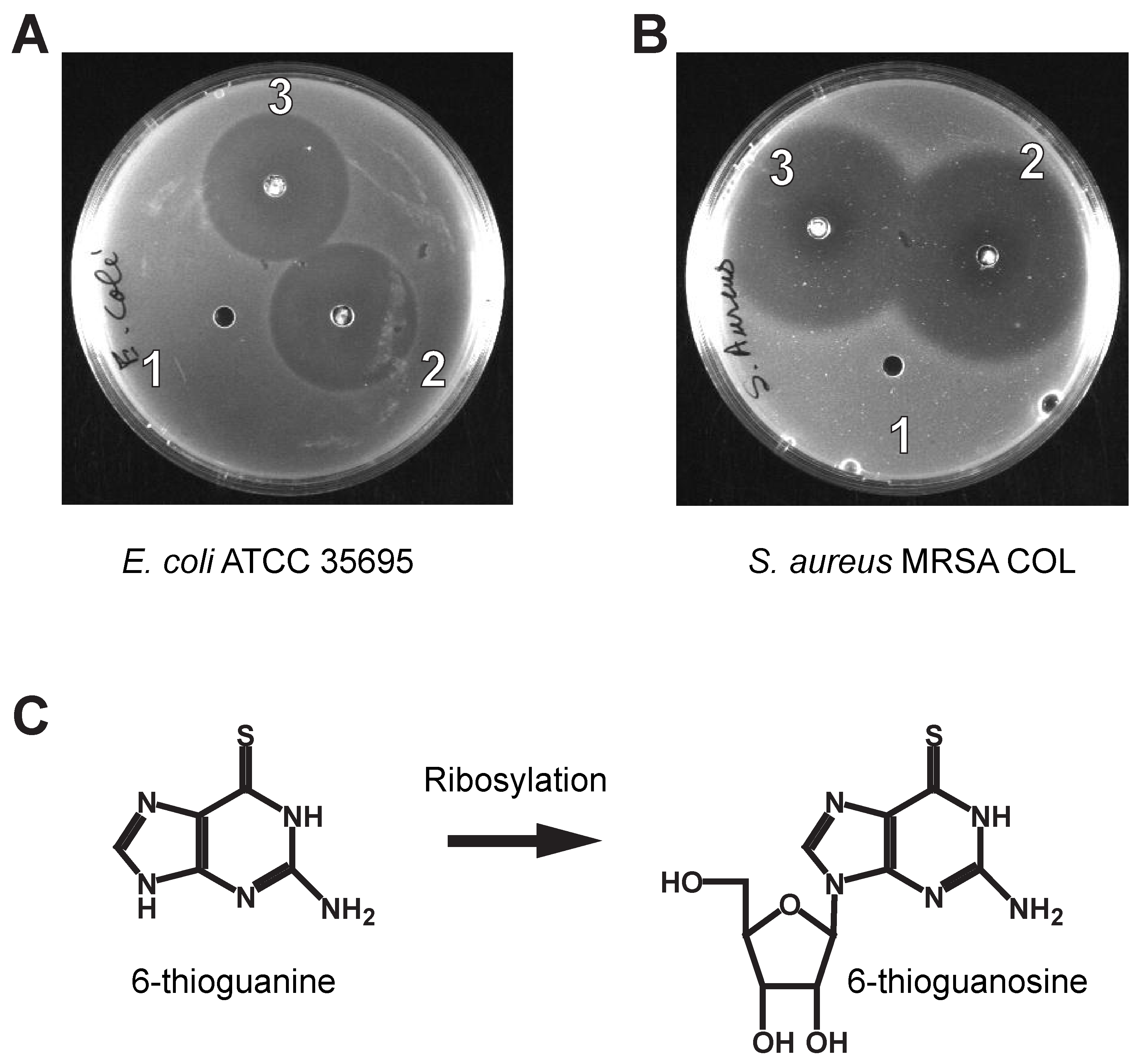

Supplement: Figure S7 — Antibiograms performed on strains of E. coli ATCC 35695 and Methicilin Resistant S. aureus strain COL. E. coli ATCC 35695 (A) and S. aureus strain COL (B) were grown in absence (well #1) or in presence of 7.5 µg (well #2) or 15 µg 6-thioguanine (well #3). Please note that 6-thioguanine is able to be ribosylated (C) and incorporated in DNA[40], which probably explains its riboswitch-independent antibiotic activity toward both E. coli and S. aureus. (3.01 MB TIF) [file ppat.1000865.s007.tif]

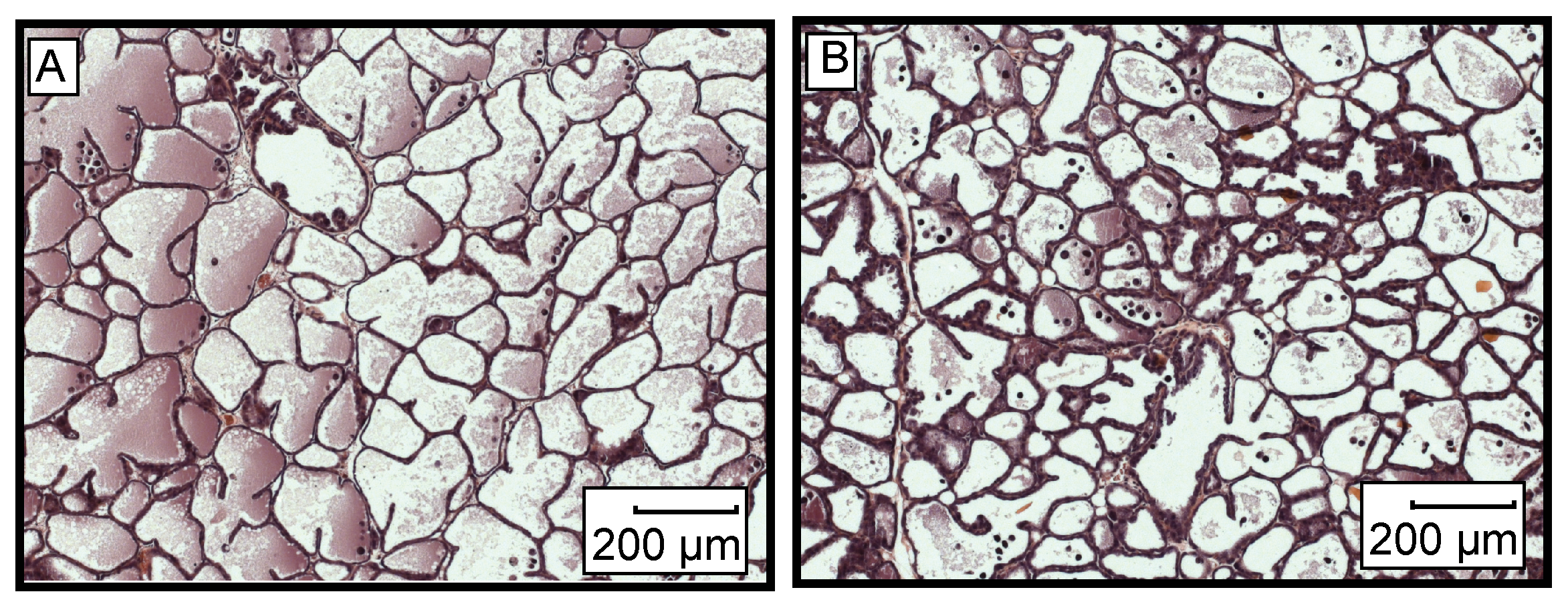

Supplement: Figure S8 — Histology of mice mammary glands treated with PC1. Mice were either injected with (A) 100 µL PBS or with (B) 100 µL PBS containing 100 µg of PC1. The treatment was allowed for 6 h and mammary glands were excised, fixed in 4% formaldehyde for 24 h at room temperature and embedded in paraffin wax. Hematoxylin-eosin staining was done on sections of 5 µm thickness. Magnifications on the pictures are 200×. The histology study reveals that there is no observable damage to the gland following PC1 injection. (5.90 MB TIF) [file ppat.1000865.s008.tif]
